# Assessing the catchment area of German comprehensive cancer centers by merging geographic healthcare data – an initiative of the ONCOnnect consortium

**DOI:** 10.1007/s00432-025-06272-0

**Published:** 2025-09-10

**Authors:** A. Kerscher, M. Metzler, J. Kapitza, L. Bernhardt, K. Dedner, M. Krebs, C. H. Brandts, on behalf of the ONCOnnect Consortium

**Affiliations:** 1 Comprehensive Cancer Center Erlangen-EMN, Erlangen, Germany; 2 Bavarian Cancer Research Center (BZKF), Erlangen, Germany; 3 Comprehensive Cancer Center Mainfranken, Würzburg, Germany; 4 Medical Informatics, Friedrich-Alexander-Universität Erlangen-Nürnberg, Erlangen, Germany; 5 Division of Pediatric Oncology and Hematology, Department of Pediatrics and Adolescent Medicine, University Hospital, Erlangen, Germany; 6 University Cancer Center (UCT) Frankfurt, Goethe University, University Hospital Frankfurt, Frankfurt am Main, Germany; 7 Comprehensive Cancer Center Augsburg, Faculty of Medicine, University of Augsburg, Augsburg, Germany; 8 Department of Medicine, Hematology/Oncology, Goethe University, University Hospital Frankfurt, Frankfurt am Main, Germany; 9 National Center for Tumor Diseases (NCT), NCT WERA, a partnership between DKFZ, and the Universities and University Hospitals in Würzburg, Erlangen, Regensburg and Augsburg, Germany

## Synergizing data from the national cancer centre certification system with the ONCOnnect consortium of the german comprehensive cancer centers

Ensuring fair and equal patient access to state-of-the-art cancer care is a major challenge for clinicians and policymakers. With an aging population and a fractured health care system, innovative approaches are required to prevent increasing disparities in urban and rural areas of Germany. Efforts to reduce these potential disparities and to provide access to innovation are implemented either as top-down or bottom-up approaches (Illert et al. 2023). The German Cancer Aid (Deutsche Krebshilfe) is supporting and funding several bottom-up strategies through its network of Comprehensive Cancer Centres (CCCs) (Brandts 2017). Currently, 14 CCCs and CCC consortia (of two or more university partners) have formed a CCC network, covering 26 university CCC sites across Germany.

In a competitive CCC application and renewal process, the German Cancer Aid ensures strict quality criteria in various areas of cancer care, including translational research, clinical trial activity, supportive care and precision oncology (Westphalen et al. 2020). Besides certification as an “Oncology Center” (by the German Cancer Society) (Kowalski et al. 2017), CCC must demonstrate a patient-centred approach in all its activities and provide extensive education initiatives to train the next generation of cancer experts. Importantly, German CCCs are required to establish an efficient outreach network that ensures close collaboration with regional healthcare providers (Brandts 2019). These funding requirements together with certification needs have significantly improved access to high-quality cancer care across Germany (Schmitt et al. 2023; Griesshammer et al. 2023).

Recently, the ONCOnnect consortium was formed to harmonize, synergize and support outreach activities across all CCCs (funded by the German Cancer Aid, www.ccc-onconnect.de). As part of ONCOnnect, researchers are analysing the geographic catchment area of cancer centres to assess the cumulative coverage of the joint CCC network – an analysis that, spanning across site boundaries, represents the first step toward monitoring the success of outreach efforts of the German CCCs.

## Mapping the individual outreach of CCC sites across Germany

As of 2024, CCC and CCC consortia must provide a map of their geographical catchment area during their application process to the German Cancer Aid for obtaining CCC Network membership. For this, they can use a software tool provided by German Cancer Aid, known as the “Krebshilfe Map”, which—through harmonised guidelines for both implementation and format—produces a standardised visual that facilitates a consistent comparison of the centres during the evaluation process. This application integrates the residential postal code areas of all patients receiving cancer care at a given centre with corresponding population densities. As a result, it generates a visual representation of the catchment area based on the local proportion of cancer care provided by that centre. Importantly, the software runs locally, ensuring that patient-specific data can be analysed on premise.

**Fig. 1:**
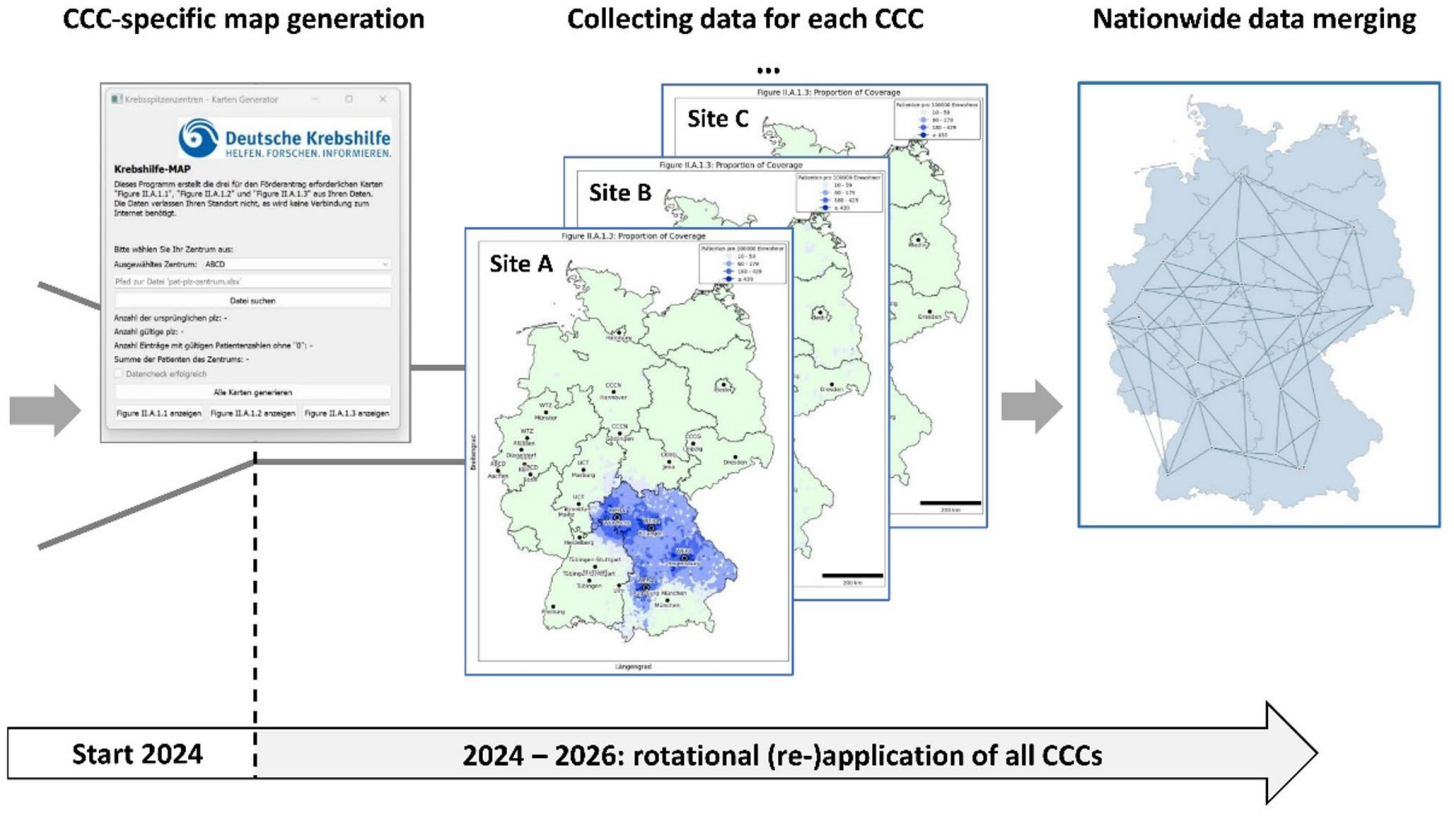
Integration of outreach data within the CCC Network. Starting with the year 2024, CCC have to provide a standardized outreach map based on site-specific cancer care data. Rotational application of all centres across Germany will provide a harmonized dataset to assess nationwide coverage

As part of the rotational application process, all CCCs will provide German Cancer Aid with site-specific maps of their catchment area. Thus, German Cancer Aid will gain valuable insights into local outreach characteristics and regional disparities in cancer care (Fig. 1).

## ONCOnnect: mapping the nationwide reach of the German CCC network

Beyond visualising differences between centres and identifying site-specific challenges to patient access, the gradual influx of harmonised outreach datasets will lay the foundation for assessing the cumulative reach of the German CCC Network. Within the ONCOnnect consortium, we plan to characterize all regions across Germany to evaluate their access to CCCs. Consequently, this will allow us to detect “white spots” – areas with limited access to innovative cancer care provided by CCC sites, as shown for access to personalized oncology approaches (Lüke et al. 2022; Krebs et al. 2024). By merging geographic healthcare data with key performance indicators such as clinical trial activity and molecular tumour board referrals, the “Krebshilfe Map” can also pinpoint underserved regions and support measures to improve patient access. For example, the findings can be used to inform evidence-based interventions, such as expanding collaborations with local oncologists and patient advocacy groups in areas with low referral rates (Krebs et al. 2024); at the same time, areas with particularly strong representation could be analysed as best-practice examples. In the long term, this approach aims to create a data-driven and more equitable oncology care landscape – ensuring access to innovations (such as clinical trials and precision oncology) regardless of geographic or structural barriers. We envision that the ONCOnnect consortium will provide a deeper understanding of local outreach determinants and contribute to improving patient access in previously underserved areas.

## Data Availability

No datasets were generated or analysed during the current study.
